# Reports of Cases of Disease; with Remarks

**Published:** 1848-04

**Authors:** John Dawson

**Affiliations:** Jamestown, Ohio


Art. III.—*Reports of Cases of Disease; with Remarks.* By John Dawson, M.D., of Jamestown, Ohio.

*Two Cases of Periodical Asthma Treated with Quinine.* Since sulphate of quinine has come into general use as a remedy in febrile and intermittent diseases, its application has been suggested to other diseases different in some of their characters, but agreeing, or being supposed to agree, in some pathological element common to them all. Among these complaints is asthma. By B. R. Hogan, U. S. A., a notice concerning the efficacy of quinine as a remedy in asthma, was published some years since in the American Medical Intelligencer. It is there stated that, from its happy influence in relieving congestion in our autumnal fevers, he was *a priori* induced to administer it in asthma. In every instance, he adds, in which it was given, the complete relief of the dyspnœa, and termination of the paroxysm succeeded in an hour. It is further stated that the paroxysms were as '*tractable*' to the influence of quinine as the slightest quotidian. John A. English, M.D., of Alabama, it seems, also used the drug in a long standing case of asthma with complete success, which had resisted the usual remedies. "Four grains of quinine operated like a charm, and relieved the patient in thirty minutes."

Published to the world, such testimony has induced various practitioners to give the article a trial in the complaint before us. Before I used it myself, I was informed by several practitioners of good observing faculties that they had made trials with it, and found it successful in arresting paroxysms of the complaint, in patients that had long been addicted to it. A physician, subject for years to very distressing attacks, for which he had used lobelia with only temporary benefit, assured me, that quinine, in tolerably large doses, had done him more good than any thing he had ever used. He related to me the case of a friend of his, who had labored under habitual attacks of a very annoying character, who also derived great benefit from quinine.

Induced by such flattering testimonials to give the article a trial, I selected some cases that were considerably characterized with the law of periodicity. These, in a brief manner, I will now detail.

Mrs. K. B., 51 years old, and never having borne any children, was attacked about fifteen years since, during the latter part of spring, with sensations of sleepiness, followed by uneasiness in the chest, which gradually ultimated in a paroxysm of asthma. Since the first attack, the complaint, as she informed me, has recurred *annually* about the same season of the year. Generally at the approach of a heavy shower of rain, attended with much thunder and lightning, the malady makes its advent. The presence of the *cirrus*, *cumulus*, or *stratus* variety of clouds, seems to produce no uneasy sensations; but no sooner does the *nimbus* or *rain-cloud* makes its appearance in the atmosphere, than she is seized with stricture of the chest and great difficulty of breathing. A paroxysm of this kind, without medicine, lasted from three to twelve hours, leaving the system in a very debilitated condition.

Three years ago, I was called to see her, and found her laboring under a severe paroxysm, which had made its appearance simultaneously with the approach of a thunder-storm. I prescribed quinine in large doses. Feeling a deep solicitude in the result of the prescription, I waited to see its effects. From the first dose I could see none. The second was followed by more or less mitigation in the symptoms; and not long after the exhibition of the third, the most urgent phenomena subsided.

Encouraged with this result, I advised the lady, the year following, to anticipate the paroxysms, by commencing the use of quinine some four or five weeks previously to their expected period of recurrence. This she did; but during the month of May she was, as formerly, again severely afflicted.

This year (May 29th, 1847,) I was requested to see her again. I found her, as previously, in great distress. In the afternoon of the above date a very dark cloud made its appearance in the west, which in a short time proved to be charged with hail that caused great destruction to the crops of grain and fruit. As this cloud approached, the difficulty of breathing and constriction of the chest were developed, and carried to such an extent as almost to produce suffocation.

Quinine was again administered in about 5 gr. doses. Relief followed, not, however, as promptly as it had done before.

Case II. M. C., a gentleman sixty years old, of a plethoric habit and with some predisposition to asthma from hereditary taint, informed me that, about twenty-four years ago, after having heated his system very much, from which he took a bad cold, he was seized with a paroxysm of asthma, for which he took medicine at the time and got bled. The constriction and difficulty of breathing passed off after a few days, and he enjoyed, as previously, very good health. At the accession of warm weather the following spring, he had another attack; and since that time he has suffered *annually* during the month of either May or June. As in the previous case, the fits have generally, if not invariably, been ushered in by the approach of a thunder-shower. After the first paroxysm has made its appearance in the spring, he enjoys but little immunity from the complaint until the weather clears up, the atmosphere gets dry, and warm weather becomes confirmed.

For the relief of these distressing fits, this gentleman got into the habit of taking nauseants, which gave him more relief than any thing he had used. At my instance, being in my office at the time with a paroxysm upon him, he took a large dose of quinine. He afterwards took several doses while the distressing symptoms were upon him, but, as far as he could see, with little or no benefit.

If this patient was not mistaken, the quinine produced rather a singular effect on his bowels. He took the article in from five to ten grain doses, and he informed me that in every instance it produced, or rather its use was followed by bloody discharges from the bowels, an affection, as I understood him, that he had never experienced before nor has felt since.

Case. III. For the following case I am indebted to Dr. Wm. H. Harper, of Lima; Ohio.

It occurred to a girl eleven years old, who had suffered from an attack of hooping-cough, which was followed by periodical paroxysms of asthma. These came on once every month, and had resisted several plans of treatment.

The treatment was commenced by Dr. Harper with cathartics, and alterative doses of blue pill. Afterwards quinine, in three grain doses, twice a day, was administered during the time that the system was free from the paroxysms. She had but one attack of the disease after this treatment was instituted, which was much lighter than what she formerly experienced. The Doctor saw the patient fifteen months after she had been under his care, and she still remained perfectly exempt from the malady.

*Remarks.*—Such cases as the above are more numerous than might be supposed. During the last five or six years I have heard of other cases, not identical, perhaps, with those above noticed, but afflicted in such a manner as to be worthy of being styled *habitual asthmatics.*

With respect to the character of the exciting cause, it doubtless differs in different cases. Those we have noticed were evidently connected very closely with the barometrical condition of the atmosphere, and also with the season of the year. When the proper time of the year approached, more or less uneasiness in the chest was experienced, but the paroxysms were not excited until the barometer indicated the approach of a heavy fall of rain.

In neither instance did the disease seem to depend in any respect on *hay*, as is supposed to be the case with what is now in England termed "*hay asthma.*" The paroxysms came on, as far as I learned, in both cases, some considerable time before the harvesting of hay in this region commenced.

After the complaint has made its advent, the period of its duration, I believe, has been but little, if any at all abridged, by any plan of treatment. Emetics, nauseants, etc. it is true, have operated as palliatives, mitigating the dyspnœa for the moment, but incapable of abridging the period of the disorder. It seemed that the complaint had a certain cycle of changes through which it had to run, and from which it could not be moved. For the accomplishment of this, a period of some two or three weeks has been required once a year since they have been first afflicted.

With the result of the quinine in these cases I was not favorably impressed. A more extensive use of the drug might have led to different conclusions. It seems that it has been used not only in our own country with good effects, but in the *Medical Gazette* of England, Dr. Gordon states that he has been completely successful in emancipating two persons from the disorder with the sulphates of quinia and iron. These had been afflicted with annual attacks of *hay asthma* for fifteen or twenty years.

Since the attention of physicians has been called to lobelia inflata as a remedy for asthma, I am of the opinion that it has shown itself to possess as much control over the pathological condition in several varieties of this malady as any other drug found in the materia medica. All experience proves that the complaint, when amenable to remedies at all, shows itself to be more controlled by something that will relax the general system, such as emetics, nauseants, etc., than anything else. The powers of lobelia to produce these effects are unquestionable, and it is on this account, without doubt, that it has obtained so much reputation as a remedy in the complaint before us. Like tobacco, it possesses the power in a preeminent degree to relax the system; but the relaxation, nausea, vomiting, etc. which it produces is not so intense. It is hence a much safer article in all those diseases where the use of either is indicated, and must take a very respectable rank as an available therapeutic agent in the hands of the practitioner.

*A Case of Diabetes Mellitus.*—L. M. consulted me in the spring of 1845, and informed me that during the year 1839 he had an attack of intermitting fever, of the quotidian type, which lasted for six months. After this passed off, he experienced some trouble in his urinary organs, which ultimated in diabetes mellitus. During a period of five years, as he informed me, he tried for medical aid some very skilful practitioners, without experiencing anything more than a palliation of some of the more urgent symptoms.

His condition when he applied to me was as follows: Age fifty years, temperament lymphatic and sanguine, frame thin and light; before contracting the disease, he weighed 165 lbs.; at the time I examined him, 150 lbs.; tongue fissured and slightly coated, with a bitterish taste in the mouth; respiration natural; pulse 112; sensations of too much warmth in the skin, but perspired very little; appetite craving; thirst very urgent; bowels costive; frequent disposition to pass urine; urine decidedly sweet and very clear, and the quantity much more than natural; felt at times inability to retain the urine. The negative symptoms were, no pain in the kidneys or any of the urinary apparatus. Prescription, muriatic acid three times a day, and aperient pills.

July 26. The patient used the acid and pills for a period of five weeks, at the end of which time his pulse was found to be from 104 to 106; skin slightly moist; thirst not so urgent; tongue still coated; something like a burning sensation that passed from the toes to the hips; desire to pass urine not so frequent; urine of a slight straw color, and still saccharine even to the taste; bowels more regular than previously; general system evidently undergoing emaciation.

Above prescription continued, and an animal diet ordered.

August 11. Pulse 100; tongue coated; taste in the mouth bitterish in the morning; thirst moderate; temperature of the skin slightly augmented; skin rather dry, though he has occasionally perspired a little; appetite not so craving; discharges from the bowels natural and regular; voids urine from seven to nine times in twenty-four hours, passing about a pint each time; urine less colored than formerly, and decidedly sweet.

September 8. At this date the patient was weighed, and it was ascertained that from the time he commenced taking the medicine he had lost in weight four and three-fourth pounds. His pulse at this time was found to have increased in frequency, being 116; thirst somewhat diminished; appetite nearly natural; tongue not as much coated as previously; chills in the evenings; urine not apparently altered in quality; about nine pints voided every twenty-four hours; free from pain, except a slight soreness in the muscles of the thighs.

Prescription, muriated tincture of iron.

November 2. Pulse 120; respiration 20; tongue slightly coated; taste bitterish; thirst still very urgent, drinks most in the evening; urinates very frequently, passing much more than the usual quantity; urine still sweet; a burning sensation along the urethra during micturition; a sensation of weakness and relaxation after taking food.

The weight of the patient at the above date was 134 lbs.

December 11. Finding the treatment with the acid and iron to be of little or no service, I ordered one drop of creosote in mucilage three times a day. At the time of this prescription the weather was cold—a circumstance that made the patient worse than previously; his appetite had failed; his muscular strength was impaired; pulse not increased in frequency; a cough had made its appearance; the feet and legs were considerably swollen, and the bowels costive. The patient died after having used the creosote for ten or twelve days.

*Remarks.*—The disease in this case in running its course occupied a period of between six and seven years. During the greater part of this time the patient, as he informed me, voided urine in excess. The quantity discharged at different periods varied with the different kinds of diet used, the circumstances by which he was surrounded, medical treatment, etc. Throughout the whole period, the color of the urine was more transparent than natural, being occasionally as clear as common well-water. Sometimes the odor was considerable, at other times slight. The amount of sugar contained in the urine was never at any one time ascertained. After the case came under my care, I sent a specimen of the urine to a chemist to have it analyzed; this, however, was not done.

Constantly, from the time the patient was invaded, there was a gradual loss of flesh. This, however, was not rapid at any period during the course except during a few of the last weeks of the patient's illness. All the time while the emaciation was going on, the appetite was craving, and the amount of food consumed was much above the ordinary requirements of nature. The trouble in the digestive apparatus consisted not so much in a want of ability to perform its functions, as in a *perverted* action, by which the elements designed for nourishment were converted into sugar.

Of the remote and proximate causes of this case we ascercertained but little. It did not, as is usually thought to be true of such cases, follow the protracted use of luxurious articles of diet. The patient had for most of his lifetime been in circumstances by no means favorable to such a course of living; he was a farmer by occupation, used a farmer's fare, and labored more or less all his life. His disease, as previously remarked, followed a protracted attack of intermittent fever, and it may be that its origin was closely connected with some of the alterations in function or structure left upon the system by that complaint. Nothing in the progress of the case threw any light on its pathology. Shortly after the first attack there was more or less uneasiness in the urethra; this, however, mostly subsided, being afterwards only occasionally experienced. The symptoms did not indicate any disease in the kidneys or any other part of the urinary apparatus.

The opinion of the older authors, that the mischief is mostly confined to the urinary organs, is not so plausible as that of modern writers, who locate it principally in the digestive apparatus. Maitland, we know, found sugar in the stomach of a diabetic patient who had been living exclusively on roasted beef for three days previous to the autopsy. McGregor, we also know, has recently traced the existence of sugar in the blood. These facts go to favor the position that the malady, instead of having its seat in the kidneys, depends upon some fault in the organs of digestion—something like a perverted condition, that renders the stomach not only unable to digest sugar, but compels it to manufacture this substance out of articles that previously contained none.

From 1839 until I saw him in 1845, this patient was more or less under the influence of drugs. From the fact that during this time he had consulted a number of medical gentlemen, including, as I understood, some very eminent, I inferred that he had been subjected to the use of the most approved remedies of our elementary works; and as he had received no benefit from anything used, I concluded it a fair case for experiment. In his twenty-second Aphorism, Hippocrates observes:

"All diseases which proceed from repletion are cured by evacuation, and those which proceed from evacuation are cured by repletion, and so of the rest: contraries are the remedies of contraries." This we call in our day the antipathic or enantiopathic method of employing remedies, and the axiom adopted is, "*Contraria contrariis opponenda.*" Something similar to this method I concluded to adopt in the case before us, and consequently directed the use of muriatic acid. Under the use of this drug the patient was kept for a period of about six months. During the first two months there seemed to be a slight amendment; the quantity of water discharged was not so great, nor was it thought by the patient to be so saccharine; moreover the thirst and craving appetite were about this period somewhat moderated. But after this the patient retrograded, and notwithstanding the acid was increased to the full amount that the stomach would bear with any degree of convenience, and the dietetic regulations pretty well observed, the patient continued to retrograde, and, as a consequence, the medicine was discontinued. A short time before the patient died, I concluded to try the suggestion of Watson relative to the use of creosote. This article was taken in doses of one drop with mucilage three times a day. Owing either to the late stage of the complaint in which it was used, or perhaps to a want of efficacy in the drug itself, there was no perceptible improvement made in the disease.

At the termination of this case, disease of the lungs was developed. To what extent this existed, and the relations it had with the fatal result, we had no means of determining. It seemed probable to us that the lesions of the chest were entirely consequent upon the corrupted and impoverished condition of the circulating mass. The long confinement to which the patient was subjected, by which the ventilation of his blood was prevented, had, no doubt, a tendency to favor the circumstances necessary to the deposition of tubercles. As far as I could learn, the dietetic regulations during most of the time that this patient was afflicted, were by no means such as could be desired. Had he been subjected to Rollo's plan of living exclusively upon an animal diet, or to that of Bouchardat, of using gluten bread, (bread in which the starch has been separated almost entirely from the gluten,) the result might have been different. It is very difficult, however, to confine a patient to either of these plans. The best course, perhaps, would be to allow the patient to combine them or use them alternately.

*A Case of Ascites, resembling Pregnancy.*—On the 8th of August, 1843, I was called on to ride to the residence of Mr. S. to see his wife, who, he said, was about to be confined in childbed. Having arrived at the house, I was met by a midwife, who informed me that she was on her second visit, and that she had made an examination, and found that "the mouth of the womb had growed up." To get more of the history of the case, I turned my attention to the lady herself. She informed me that she had not been well for the last two years, but that about nine months or a year since she had been troubled with slight bearing down pains, difficulty in breathing at times, "inward weakness," and loss of appetite. Continuing for a while, these symptoms were followed by an enlargement of the abdomen and occasional sensations resembling the motions of a fœtus, and, as a consequence, she concluded that she was pregnant. When I made my examination, the attitude was that of sitting up in a half recumbent posture on the side of the bed—a position in which she had fixed herself, as I understood, for the purpose of being delivered by the midwife present. The countenance was sallow and emaciated, tongue slightly coated, taste depraved, and breath fetid; the general surface was dry, rough and shrunk, except the abdomen, which was about as much distended as was usual for her during the last period of pregnancy, and the feet and legs, which were anasarcous; the pulse was feeble and rather small; there was a nervous irritation that kept her from sleeping but little through the night; thirst pretty urgent; and bearing down pains that came on every fifteen or twenty minutes. So entirely periodical were the pains that little doubt was entertained by the midwife or friends of their being the result of parturient efforts of the uterus. Not suspecting anything but pregnancy, and being informed by the midwife that the os uteri was in an unnatural condition, I determined to make an examination per vaginam. The vagina was found relaxed and covered with an unhealthy offensive secretion. The os uteri was the seat of a scirrhous lesion, and was almost completely obliterated by the morbid growth; extremely irritable and tender to the touch, it was also the point from which radiated sharp cutting pains, which recurred periodically, and resembled to a very considerable extent the pains of labor. Placing my hand upon the abdomen, the sense of fluctuation, form of the abdominal tumor when the patient was lying on her back, and absence of what I considered the essential signs of pregnancy, clearly indicated that it was a case of *ascites* instead of pregnancy.

*Remarks.*—There was something of interest in the diagnosis of the above case. In favor of its being pregnancy, we had the abdomen to become gradually enlarged, attended with a swelling of the feet and ankles; the quickening sensations which the patient imagined herself to have experienced at the fourth or fifth month of her disease; the bearing down pains she experienced, which came on periodically, and so much resembled the pains of labor that the patient herself—who was the mother of eight or ten children, and who on this account, it might be presumed, ought to have some judgement—was entirely deceived; and lastly, the cessation of the catamenia simultaneously with the advent of the complaint. All these taken together would naturally do something towards making out, in appearance at least, a case of pregnancy.

As going to show that it was a case of ascites, we had the general form of the abdominal tumor, its elasticity, the sense of fluctuation on percussion, and, negatively, the absence of sounds corresponding to those of the fœtal heart and of the circulation of the blood in the veins and arteries distributed through the gravid uterus, (placental souffle.) Concerning the diagnostic value of the condition of the os uteri—which, as before remarked, was scirrhous—we can tell but little from our own experience in such cases. It might reasonably be supposed that conception could not take place when the os uteri was the seat of malignant disease. As a general rule, this would probably hold good; yet there are a few instances recorded where pregnancy has been complicated with malignant fungoid disease of this part. Dr. James Johnson had a case of this kind in April, 1840.

Perplexed as physicians frequently are in making out the particular character of a disease, the subject of diagnosis cannot receive too much attention. Indeed, even after great diligence assisted by long experience, many of the enlargements of the abdomen are of difficult comprehension. It is known that the celebrated John Hunter once mistook a distension of the bladder for ascites, and absolutely tapped it. Enlargements occasioned by hypertrophy of the liver, polysarcia adiposa, ovarian dropsy, etc. are once in a while well calculated to embarrass.

There is another enlargement, an example of which I witnessed in a female some years ago. While laboring under a paroxysm of hysteria, the abdomen became very much distended, stretching the fibres of the parietes to the utmost degree. While in this condition I arrived, and supposing from the great pain of which she was complaining that it was a case of pregnancy, I examined the uterus per vaginam, and found that it was in an unimpregnated condition. Before I had been in the house an hour, the distension and pain left the abdomen and seized upon the chest. Between these two cavities the swelling alternated, until the patient was relieved by purgatives and antispasmodics. The distension of the chest seemed to arise from the rushing up of the viscera of the abdomen against the diaphragm—a circumstance that made the respiration very laborious. What the trouble was which gave rise to such anomalous phenomena seemed difficult to determine. The bowels must have been distended with gaseous matter of some kind or other; and besides this there were violent spasms in the abdominal muscles and diaphragm. It is cases of this character, I presume, that, for want of a more significant name, some writers denominate "hysterical tympanitis." I learned that this woman had previously suffered from such attacks, and that she had been relieved by something like the same means that I employed.

With respect to the prognosis of the above case of ascites I was closely interrogated by the lady herself. "Is it possible, doctor, for me to get well?" she inquired. I answered, "I think, Mrs. S., that you will die." Convinced as I was from all the circumstances connected with her case that the issue would be fatal, and knowing her to be totally unprepared to die, nay, even very wickedly disposed, I could see no impropriety in giving her candidly my opinion of the result. Unfavorable prognostications—by which I presume is meant telling patients they must die, when we believe such must be the result—are thought to be not in accordance with good etiquette, for in many cases very much may be done to the advantage of the patient by invoking the influence of hope, by keeping back probable results, and exciting the mind strongly in favor of recovery. Of the correctness of this course as a general rule we have not the shadow of a doubt. Our case, however, was one of a peculiar character—a malignant disease of the os uteri complicated with dropsy, making together a set of lesions of a character not at all ambiguous as regarded the final result. When such proves to be the case—when, indeed, we are satisfied that it is impossible that the body can be saved—what impropriety is there in making patients acquainted with their condition, that, if possible, they may have time to make an effort to save the soul? After I informed Mrs. S. of her condition, she became penitent, and until her death constantly implored the forgiveness and mercies of her Creator.

*A Case of Purpura Hæmorrhagica, following the protracted Use of Sulphur.*—The subject of this case was a boy nine years old. He had been taking sulphur for some purpose or other, and becoming fond of it, used a considerable quantity without the knowledge of his parents. While engaged in this practice, he went into the water and bathed. Swelling of the extremities, stiffness of the joints, with chills and fever, followed. For the relief of these things he took emetics, cathartics and diaphoretics, and was better for a few days. Exposing himself again, however, fever supervened, followed by periodical pains in the bowels and vomiting of blood. A clyster brought away a portion of coagulated blood from the bowels, after which the periodical pains measurably ceased. The patient was then put on the use of quinine and continued taking it for two days in small doses. The pains in the bowels and bloody discharges returned while taking the quinine, and deep purple spots made their appearance on various parts of the body, together with strong symptoms of anæmia and general debility. After the occurrence of these symptoms, the patient was put upon the use of sulphuric acid and quinine in proper doses, with an occasional dose of Peruvian bark and rhubarb. An amendment was quite obvious for several days. The periodical pain, however, which was previously experienced in the bowels, seated itself in the head, the urine became highly colored, depositing a dark sediment, and the feet and face grew edematous. Quinine dissolved in sweet spirit of nitre was then given in proper doses, and the patient was ordered to be bathed in a decoction of oak bark. Under the use of the nitre and quinine he improved and ultimately regained his health. The bathing in the oak bark decoction was discontinued in consequence of its producing febrile excitement.

*Remarks.*—The cause of the disease in the case under consideration is perhaps not easily solved. It was attributed by the friends, and perhaps rightfully, to the sulphur taken. That sulphur is capable of producing important alterations in both the solids and fluids is evident from the fact, that silver articles become discolored in the pockets of persons who have been for any considerable time under its influence; and it is also known that the odor of hydrosulphuric acid is communicated to the sweat, urine and milk of persons who have been taking it for any length of time. By the German physicians it is called a resolvent, and by a late English writer of great distinction it is classed with liquefacients, which signifies medicines that are supposed to check the solidifying while they promote the liquefying processes in the animal economy; they loosen and soften texture, and in this way it is supposed that they assist in the removal of adhesions and exudations. This effect is strikingly seen in the use of mercurials, which belong to the same class, in the removal of deposits of coagulable lymph, glandular indurations, and enlargements and thickening of membranes—as of the periosteum, for example—and also very strikingly in its power of rendering the gums spongy.

Considered, then, as possessing liquefacient properties similar to those of mercury, antimony, etc., we can see no good reason why it may not produce those alterations in the blood which characterize purpura hæmorrhagica. Reasoning *a priori*, this would be the only conclusion to which we could come. We would suppose that it would diminish, if taken for any length of time, the normal quantity of the solid constituents, the blood corpuscles and the fibrin, while it would relatively increase the quantity of water. In the only case that we know of in which the blood was analyzed, Routier found 1000 parts of the blood to contain: water, 795·244; solid constituents, 204·756; fibrin, 0·905; blood corpuscles, 121·701; residue of serum, 83·405.

Although from this analysis the blood in purpura does not appear to be identical in composition with what obtains in spanæmia, it is nevertheless decidedly poor—a condition which has induced Simon to class purpura with such diseases as anæmia, hydræmia, carcinoma, scrofulosis, chlorosis, scorbutus, etc.

*A Case of Dengue terminating in Gangrena Ossium.*—During the years 1841-2 dengue—or, as it is popularly termed, '*saint measles*'—prevailed here as an epidemic, affecting all classes of persons, and producing very much distress, but generally attended with slight mortality. The symptoms chiefly entitled to the character of pathognomonic were severe pains about the articulations at the commencement and during the course of the malady, an anomalous eruption on the skin, and intolerable soreness of the muscular system; inflammatory fever was constantly associated with these. As a general rule, the sequelæ were neither numerous nor grave. One exception occurred, however, and our design in reporting it is the hope that it may assist us in arriving at proper conclusions concerning the pathology of dengue. The case to which we allude is that of

M. T., aged sixteen years, of rather corpulent habit and good general health previous to contracting the disease. In the month of February, 1843, he was taken sick, and being called to see him, we recognised his disease as being dengue. The febrile orgasm was of the synochus grade, attended with the usual symptoms, such as headache, thirst, dry skin, coated tongue, etc. The arthropathic affections were very violent, but as far as we noticed there was no eruption on the skin. The usual remedies for such indications were used, together with a decoction of eupatorium perfoliatum, a remedy of considerable reputation with the people of the neighborhood for the disease. More persistent than usual, the malady seemed to leave the tissues about the joints, and seat itself upon the bones in various parts of the system. After much suffering from this cause, caries made its appearance and has continued until the present time. It was first developed on the inferior extremities, afterwards on the scapula and the molar bone on the left side of the face. At the present time the shaft of the right thigh bone is the principal seat of diseased action. Sooner or later after the complaint became seated on any of the bones, sinuses were established, leading from the soft parts down to the seat of disease, from which sanies of an offensive character issued.

In the family of which this young man is a member there has never been, as far as I could learn, any hereditary predisposition to any of the diseases supposed to favor the occurrence of caries; and the young man himself, as has already been remarked, was healthy until he contracted the malady which has resulted in his present affliction.

From the course of this case we suppose it might reasonably be inferred that dengue in its onset and progress affects tissues closely allied to the osseous. Indeed, the arthropathic affections present in every case warrant this conclusion. Bones of a spongy texture, *cœteris paribus*, such as the vertebræ, astralagus, and other members of the tarsus and of the carpus, the sternum, and the heads of the long bones, are more liable to attacks of caries than those that are compact. In the case before us, the malady seated itself and spent its principal force upon the most compact portions of the bones, and showed no preference for those of a decidedly spongy texture.

The parts originally attacked by the carious affection have now healed up, while new ones on other bones or on other parts of the same bone have made their appearance. The general system at present, however, seems in a condition to promise ultimate recovery.

February, 1848.

